# AI-empowered imagery writing: integrating AI-generated imagery into digital mental health service

**DOI:** 10.3389/fpsyt.2024.1434172

**Published:** 2024-07-22

**Authors:** Chao Hu, Zhicheng Lin, Ning Zhang, Li-Jun Ji

**Affiliations:** ^1^ Department of Psychology, School of Education, Guangzhou University, Guangzhou, Guangdong, China; ^2^ Department of Psychology, University of Science and Technology of China, Hefei, China; ^3^ School of Public Health and the Second Affiliated Hospital, Zhejiang University School of Medicine, Hangzhou, Zhejiang, China; ^4^ Department of Psychology, Queen’s University, Kingston, ON, Canada

**Keywords:** digital mental health service, AI-generated imagery, art therapy, wisdom therapy, mental imagery, expressive writing

## Introduction

As mental health systems grapple with soaring demands, rising costs, and accessibility barriers, increasing the reach and impact of psychological therapies has become an urgent public health priority (1). Recent generative artificial intelligence (AI) advancements offer a pathway toward scalable, accessible, and potentially more effective mental health solutions. For example, AI chatbots such as ChatGPT have been proposed as a first aid for young adults with mental health issues (2). Although the value of digital mental health services has been recently recognized—due to the low-threshold access, geographic independence, constant availability, and potentially lower cost (1, 3)—the potential of generative AI remains underexplored, particularly concerning AI-generated imagery. Mental imagery, especially emotional mental imagery, is central to many mental disorders and the associated psychotherapeutic interventions (4, 5). To explore the potential of incorporating AI-generated imagery into mental health services, we propose a novel paradigm termed “AI-empowered imagery writing” (AIW).

**Figure 1:**
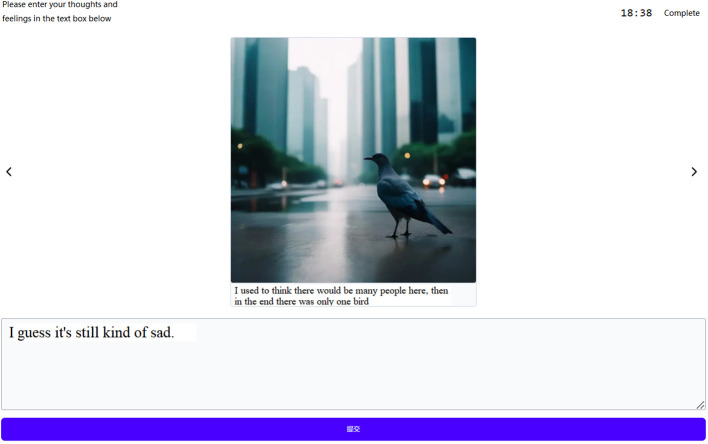
Online AIW system generated image corresponding to the writing: “I used to think there would be many people here, then in the end there was only one bird/我曾经认为这里会有很多人, 然后最后只有一只鸟”.

In AIW, as users compose sentences, the AI system generates corresponding art imagery—potentially resembling iconic art styles like Impressionism (see **Figure 1** for an example)—and adapts this imagery to reflect the emotional context of the text. This approach builds on the well-established benefits of expressive writing (EW), a cost-effective intervention that improves mental well-being and alleviates trauma-related symptoms (6–8). EW allows writers to gain valuable insights into their thoughts and feelings, particularly concerning adverse experiences. AIW enhances EW by adding a visual dimension. Like an architect employing a 3D model to visualize a building design better, AIW enables users to vividly “see” and “revise” their thoughts through generated images.

With the proper instructions from professional psychologists and psychotherapists, AIW could provide effective psychological interventions or psychotherapy for participants. For example, psychotherapists can adapt the classical instructions of EW (6, 7) and then display them on patients’ personal computing devices at home via the internet: “Find a time and place where you won’t be disturbed. Promise yourself that you will write for a minimum of 15 minutes a day for at least 3 or 4 consecutive days. Once you begin writing, write continuously … You can type on a computer, a tablet, or a smartphone. If you cannot type, you can also use audio input.” Participants can write about something they are thinking or worrying about too much, something they are dreaming about, something they feel is affecting their lives in an unhealthy way, or something they have been avoiding for days, weeks, or years.

**Figure 1** is a screenshot of our online AIW platform. The user writes in a text box, presses the return key or the “submit” button, and then AI generates the corresponding image in 5 seconds and displays it on the top of the text box. Meanwhile, the user can continue to write, submit, and see the generated images. In the following, we suggest key mechanisms that position AIW as a promising and attainable digital intervention for mental health improvement.

## Vicarious empathy and emotional expression

AIW can provide “vicarious empathy”. While it is well-known that AI can create images corresponding to textual input—capabilities offered by software like Dall-E, Stable Diffusion, and Midjourney—it is less commonly recognized that AI can also discern emotions embedded within the text, as evidenced in large language models such as ChatGPT and Claude. Leveraging these capabilities, AIW can customize imagery to align with the writer’s emotional state during the writing process. For instance, the imagery can be adjusted to be of darker or brighter hues and colder or warmer color tones. These emotionally congruent images may serve as positive reinforcement, just as empathy is rewarding in interpersonal interaction (9). This reinforcement could encourage further participation in the writing process.

AIW can even help users express their emotions. For EW to be effective for individuals who have experienced traumas, it would be better for participants to express emotions related to their negative experiences (7, 10). However, some individuals may struggle to express such feelings due to their personality traits, sociocultural factors (11, 12), or mental disorders (e.g., alexithymia) (13). Such individuals may omit emotional expression when recalling negative experiences, instead writing only facts related to the event. In this case, AI-empowered imagery may help individuals express their negative emotions by visualizing these feelings.

## Promoting wisdom

AIW has the potential to foster individuals’ wisdom (i.e., profound and comprehensive understanding of one’s experience; 14–16), thereby ameliorating emotional distress and promoting growth following negative life events. Improving wisdom is helpful for individuals who have experienced major yet commonplace adverse events (e.g., job loss, relationship dissolution, or the death of a loved one; 17, 18). Central to the construct of wisdom is perspectival metacognition (PMC), a nuanced form of metacognition that encompasses non-propositional elements like epistemic humility and the consideration of diverse perspectives (19). PMC enables a more mature and balanced understanding of events, harmonizing potentially conflicting interests and perspectives.

By incorporating the principles of PMC, AIW has the potential to facilitate metacognitive shifts and offer alternative perspectives through generated imagery. Specifically, AIW can help users transition from being immersed in their subjective experiences to observing them from an external perspective—a shift crucial for mental health and wisdom (19, 20). Moreover, the multi-dimensional imagery generated by AIW, which encompasses length, width, height, and time, may offer a more robust paradigm for metacognition than text alone. Providing richer metacognitive opportunities may be particularly advantageous when individuals write about negative autobiographical experiences, as suggested by previous clinical research (4, 5).

AIW can further facilitate wisdom development by encouraging individuals to process negative life events critically. While wisdom development often necessitates challenging “exploratory processing” such as meaning-making, this can be daunting during stressful times (15, 21). Individuals may default to “tunnel vision”, focusing solely on immediate needs and neglecting alternative perspectives (21, 22). However, AIW’s generated imagery catalyzes PMC: by externalizing the “self” to the imagery, AIW could create a psychological distance between the self and the negative experiences, thereby encouraging a more objective and expansive understanding (cf. 19, 23–25). Additionally, AI-generated imagery can be designed to include other characters from the narratives, enabling writers to adopt a more comprehensive and nuanced view of the situation, much like how schematic diagrams assist in mathematical problem-solving (26).

As writers articulate their thought process and subsequently review their text and the corresponding AI-generated images, they could gain heightened metacognitive sensitivity, which is beneficial for counteracting overgeneralized self-related thinking, a cognitive distortion linked to depression (27, 28). AIW can produce abstract imagery in response to overgeneralized thoughts, such as “I am useless”, which contrasts sharply with the more concrete and detailed imagery generated for specific life scenarios, like “I am skilled/unskilled at cooking fish.” Therapists can remind the patients of this visual discrepancy and encourage writers to engage in more nuanced narrative construction, thereby mitigating the over-general autobiographical memory that predicts depression (28, 29).

AIW can further promote wisdom development by utilizing visual art to foster new perspectives and interpretations of written content (30). Drawing on the creativity-enhancing potential of art (31), imagery from AIW can catalyze creative self-expression, a cornerstone of art therapy shown to improve mental well-being (32–34). Notably, these AI-generated images are not mere replicas of the writer’s mental imagery but rather nuanced variations. For example, in text-to-image AI software, the AI adapts existing images related to the text’s keywords, essentially offering alternative perspectives on the writer’s narrative (35, 36). Engaging with these images can be likened to getting a “second opinion” on one’s experiences, facilitating a broader perspective of these events. This aligns with research showing that a third-person perspective enhances wise reasoning during social conflicts (37) and that writing about adverse experiences from a third-person perspective (cf. first-person perspective) can improve positive emotions (38).

## Discussion

AIW presents an exciting avenue for enhancing mental health by synergistically combining fundamental elements of expressive writing, art therapy, and wisdom therapy—such as meaning-making, creative expression, and perspectival metacognition. As AI technology continues to evolve, AIW can be increasingly customized to meet individual needs, thereby optimizing the impact of writing and AI-generated art on psychological well-being.

AIW holds great promise as an attainable mental health intervention. Traditional therapeutic settings often need trained clinical psychologists and counselors. AIW, accessible via personal computing devices, offers a resource-efficient alternative that can be widely deployed at home and in public service settings like hospitals and schools. Notably, the platform can maintain user anonymity, allowing individuals to engage in therapeutic writing without fear of privacy infringement. Users can selectively share portions of their writing with the AI, receiving personalized feedback.

This emerging field raises many intriguing questions about efficacy, implementation, and potential risks. Much empirical work is needed to verify the efficacy of AIW and identify optimal implementation strategies to maximize its therapeutic efficacy and minimize its risk. For example, researchers can conduct a randomized controlled trial (RCT) to test if individuals are gaining “metacognitive sensitivity” by using AIW.

Additional questions also arise regarding the impact of AIW on writers’ mental visualization skills, which are important for mental health (4, 5). For example, could AIW enhance or impair an individual’s ability to mentally visualize and conceptualize past events? Furthermore, what are the long-term implications of such changes in mental visualization skills on overall mental health? While AIW could be particularly beneficial for those struggling with mental imagery by allowing them to conceptualize their experiences more vividly, it may inadvertently limit those already possessing robust visualization skills. To mitigate this, AIW could employ deliberately blurred or abstract images, thereby avoiding the “formatting” or “framing” of an individual’s imagination. Such an approach would preserve the interpretive space necessary for mental visualization, offering a more nuanced and flexible therapeutic tool.

Application of AIW must adhere to stringent ethical standards, transparency, respect for patient rights, data protection, and particular attention to vulnerable demographics (e.g., individuals with posttraumatic stress disorders), as recommended by e-mental health experts (1). For example, some AI-generated images may be distracting or even offensive to users. To mitigate this risk, users could provide feedback to the AI system at the beginning of writing. Users might relay their comfort level with the images and express how motivating they find the imagery for their writing endeavors. Subsequently, the system could iteratively fine-tune itself to align with each user’s emotional and cognitive preferences.

## Limitation

Empirical data are needed to test our proposal, especially regarding the long-term effects of AI-generated imagery on mental health. In addition, potential biases and limitations of AI-generated imagery across different social groups (e.g., the poor and the disabled) and cultures need to be evaluated. Researchers from diverse cultures and fields (e.g., psychiatry, computer science, sociology, creative writing, art) should collaborate to better manage the risks and benefits of AI-generated imagery (39).

## Conclusion

AI-generated imagery technology can potentially aid digital mental health services. AIW harnesses the power of AI to boost the effectiveness of expressive writing, bringing images to insights and pixels to perspectives. As this fusion develops, we must remain vigilant, guarding against risks while maximizing benefits for mental health. With thoughtful development, AIW can potently improve mental health and well-being at scale.

## Author contributions

CH: Conceptualization, Visualization, Writing – original draft. NZ: Writing – review & editing. ZL: Writing – review & editing. LJ: Funding acquisition, Writing – review & editing.
